# The Effects of Selective Hematopoietic Expression of Human IL-37 on Systemic Inflammation and Atherosclerosis in LDLr-Deficient Mice

**DOI:** 10.3390/ijms18081672

**Published:** 2017-08-09

**Authors:** Geerte Hoeke, P. Padmini S.J. Khedoe, Janna A. van Diepen, Karin Pike-Overzet, Britt van de Ven, Nadia Vazirpanah, Isabel Mol, Pieter S. Hiemstra, Frank J.T. Staal, Rinke Stienstra, Mihai G. Netea, Charles A. Dinarello, Patrick C.N. Rensen, Jimmy F.P. Berbée

**Affiliations:** 1 Department of Medicine, Division of Endocrinology, Leiden University Medical Center, Post Zone C7Q, P.O. Box 9600, 2300 RC Leiden, The Netherlands; G.Hoeke@lumc.nl (G.H.); P.P.S.J.Khedoe@lumc.nl (P.P.S.J.K.); brittvandeven@gmail.com (B.v.d.V.); N.Vazirpanah@umcutrecht.nl (N.V.); I.M.Mol@lumc.nl (I.M.); P.C.N.Rensen@lumc.nl (P.C.N.R.); 2 Einthoven Laboratory for Experimental Vascular Medicine, P.O. Box 9600, 2300 RC Leiden, The Netherlands; 3 Department of Pulmonology, Leiden University Medical Center, Leiden P.O. Box 9600, 2300 RC Leiden, The Netherlands; P.S.Hiemstra@lumc.nl; 4 Department of General Internal Medicine and Radboud Center for Infectious Diseases, Radboud University Nijmegen Medical Center, 6525 HP Nijmegen, The Netherlands; Janna.vanDiepen@radboudumc.nl (J.A.v.D.); Rinke.Stienstra@radboudumc.nl (R.S.); Mihai.Netea@radboudumc.nl (M.G.N.); cdinare333@aol.com (C.A.D.); 5 Department of Immunohematology and Blood Transfusion, Leiden University Medical Center, P.O. Box 9600, 2300 RC Leiden, The Netherlands; K.Pike-Overzet@lumc.nl (K.P.-O.); F.J.T.Staal@lumc.nl (F.J.T.S.); 6 Division of Human Nutrition, Wageningen University, 6708 PB Wageningen, The Netherlands; 7 Department for Genomics & Immunoregulation, Life and Medical Sciences Institute (LIMES), University of Bonn, 53113 Bonn, Germany; 8 Department of Medicine, University of Colorado, Aurora, CO 80045, USA

**Keywords:** atherosclerosis, inflammation, interleukin-37, hyperlipidemia

## Abstract

The human cytokine interleukin (IL)-37 has potent anti-inflammatory capacities, and hematopoietic cell-specific transgenic overexpression of IL-37 in mice protects against septic shock and colitis. In the present study we investigated the effect of hematopoietic expression of IL-37 on atherosclerosis development under low-grade inflammatory conditions. Low-density lipoprotein receptor (LDLr)-deficient mice were lethally irradiated and transplanted with bone marrow from IL-37-transgenic or control wild-type mice and fed a Western-type diet (WTD; 1% cholesterol) for eight weeks. Metabolic and inflammatory parameters were monitored and atherosclerosis was assessed in the aortic valve area. Hematopoietic IL-37 expression did not influence body weight, food intake and plasma cholesterol levels during the study. Plasma soluble E-selectin levels were increased with WTD-feeding as compared to chow-feeding, but were not influenced by IL-37 expression. IL-37 expression reduced the inflammatory state as indicated by reduced white blood cell counts and by reduced basal and lipopolysaccharide-induced cytokine response by peritoneal macrophages ex vivo. IL-37 expression did not influence the atherosclerotic lesion area. Lesion composition was marginally affected. Smooth muscle cell content was decreased, but macrophage and collagen content were not different. We conclude that under low-grade inflammatory conditions, hematopoietic IL-37 expression reduces the inflammatory state, but does not influence atherosclerosis development in hyperlipidemic LDLr-deficient mice.

## 1. Introduction

Atherosclerosis is a chronic inflammatory condition characterized by the progressive accumulation of various immune cells within the arterial wall [1]. Macrophages are very important players in the development of atherosclerosis. In addition to accumulating lipids, they are also known to display large plasticity in function ranging from anti- to pro-inflammatory phenotypes depending on specific stimuli within the atherosclerotic plaque [2]. Extensive research has focused on the characterization of pro-atherogenic cytokines and receptors that propel atherosclerotic lesion progression (reviewed in [1]). However, only a limited number of studies have investigated the atheroprotective effect of anti-inflammatory cytokines, and their therapeutic potential as immunosuppressive mediators during atherosclerosis development [3]. So far, the anti-inflammatory mediators interleukin (IL)-10 [4], transforming growth factor (TFG)-β [5], IL-33 [6] and IL-1 receptor antagonist (IL-1ra) [7] have shown to exert marked anti-atherosclerotic and atheroprotective activities in mice.

The anti-inflammatory cytokine IL-37, formerly known as IL-1 family member 7 (IL-1F7), emerged as an endogenous suppressor of innate inflammatory and immune responses [8]. IL-37 belongs to the IL-1 family that also includes e.g., IL-1α IL-1β, IL-1ra, IL-18 and IL-33. In humans, various tissues and cell types including blood monocytes [9], endothelial cells [10], adipocytes [11] and epithelial cells [12] express IL-37. However, a murine homologue has not been discovered yet. The IL-37 mRNA transcript contains an instability component that is stabilized upon the exposure of the cells to inflammatory stimuli [13] driving increased IL-37 protein expression levels in response to e.g., Toll-like receptor agonists, IL-1β and TNF-α [8]. The potency of IL-37 to suppress inflammation is evident from in vitro experiments in which IL-37 induction markedly reduced the expression of pro-inflammatory cytokines and chemokines in monocyte and macrophage cell lines [8,14].

IL-37 consists of five isoforms (i.e., IL-37a–e), of which IL-37b is most abundant [9]. Mice with transgenic expression of human IL-37b (IL-37tg) were protected from lipopolysaccharide (LPS)-induced septic shock and showed reduced tissue and systemic levels of inflammatory cytokines in response to LPS [8] and Concanavalin A-induced hepatitis [15]. In addition, IL-37tg mice were protected from dextran sodium sulfate (DSS)-induced colitis as indicated by strongly reduced inflammation and colonic infiltration of leukocytes, including macrophages. Interestingly, wild-type (WT) mice reconstituted with bone marrow from IL-37tg mice were also protected from DSS-induced colitis, indicating that hematopoietic IL-37 expression is sufficient for this protective effect [16].

These data suggest that IL-37 expression in hematopoietic cells may reduce the local influx of cells and the secretion of pro-inflammatory cytokines in inflammatory diseases, including atherosclerosis. This is further supported by a recent study in which systemic IL-37 treatment protected from atherosclerosis via modulating immune cell responses [17]. Therefore, the aim of the present study was to evaluate whether transgenic expression of IL-37 in hematopoietic cells inhibits the accumulation of macrophages in the plaque and is sufficient to protect against atherosclerosis progression. We transplanted bone marrow from either WT or IL-37tg mice into LDL receptor-deficient (*Ldlr*⁻^/^⁻) mice and assessed metabolic and inflammatory parameters as well as atherosclerotic lesion size and composition. Mice were fed a Western-type diet (WTD) containing 1% cholesterol to induce hyperlipidemia and low-grade systemic inflammation. We demonstrate that hematopoietic expression of IL-37 moderately reduced the inflammatory status of hyperlipidemic *Ldlr*⁻^/^⁻ mice, but atherosclerotic lesion size or macrophage content of the plaque remained unchanged.

## 2. Results

### 2.1. Hematopoietic IL-37 Expression Does Not Affect Metabolic Parameters

Human IL-37 was selectively expressed in the hematopoietic cells, including macrophages, of atherosclerosis-prone *Ldlr*⁻*^/^*⁻ mice by the transplantation of bone marrow cells from IL-37tg mice. As a control, *Ldlr*⁻*^/^*⁻ mice were transplanted with bone marrow from WT mice. After a recovery period of nine weeks on regular chow diet, hyperlipidemia was induced by feeding the mice a high-cholesterol Western-type diet (WTD) enriched (containing 1% cholesterol) for eight weeks. Bone marrow of *Ldlr*⁻*^/^*⁻ mice transplanted with IL-37tg bone marrow cells expressed human IL-37 mRNA at the end of the study, whereas it was undetectable in the WT-transplanted mice (data not shown), confirming successful hematopoietic reconstitution.

**Figure 1:**
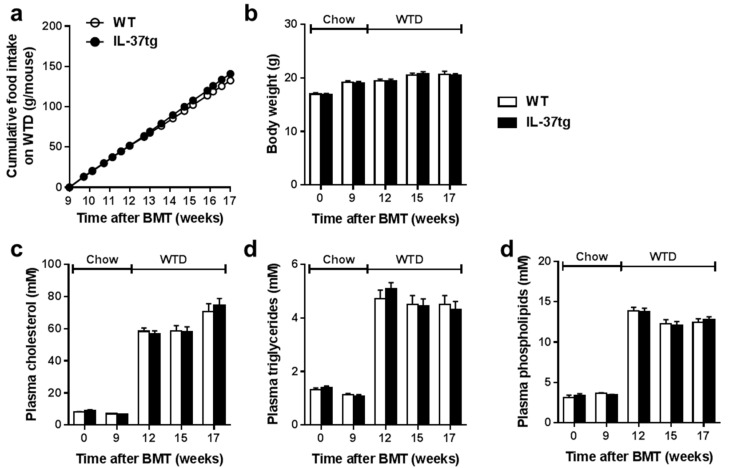
Successful expression of human IL-37 in hematopoietic cells does not affect metabolic parameters. *Ldlr⁻/⁻* mice were transplanted with bone marrow from IL-37tg or control wild-type (WT) mice. After nine weeks of recovery on regular chow diet, mice were fed a Western-type diet (WTD) for eight weeks. Cumulative food intake (**a**) body weight (**b**) and 4-hour-fasted plasma levels of cholesterol (**c**) triglycerides (**d**) and phospholipids (**e**) were monitored during the study. Data are expressed as means ± standard error of the mean (SEM); *n* = 14–15 per group. BMT, bone marrow transplantation.

We assessed the effect of hematopoietic IL-37 expression on metabolic parameters during the study. Hematopoietic IL-37 expression did not influence cumulative food intake during the recovery period (not shown) or during WTD-feeding (Figure 1a). Body weight was also not different between the groups (Figure 1b). Furthermore, hematopoietic IL-37 expression did not affect the plasma total cholesterol (Figure 1c), plasma triglyceride (Figure 1d) or plasma phospholipid (Figure 1e) concentrations during the study.

### 2.2. Hematopoietic IL-37 Expression Moderately Reduces the Inflammatory State

**Figure 2:**
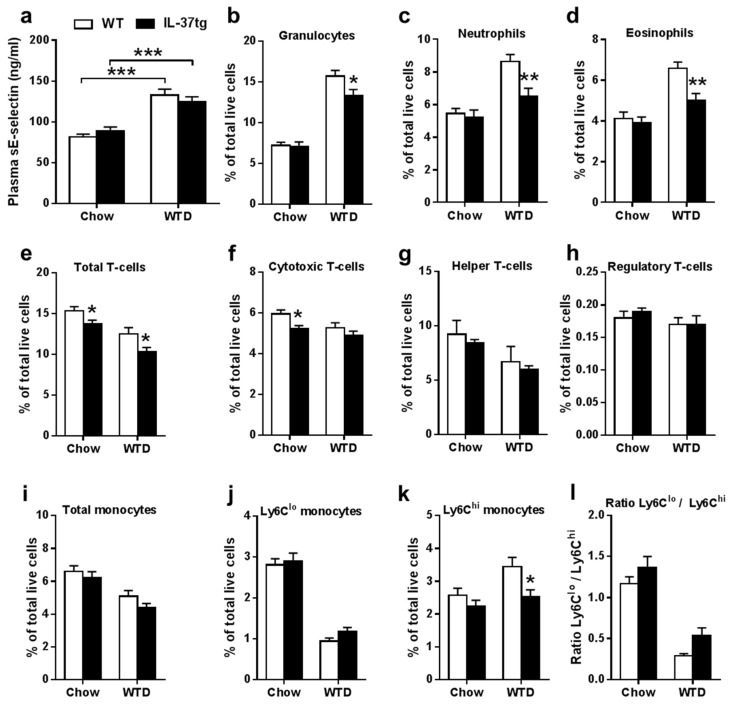
Expression of IL-37 in hematopoietic cells reduces circulating immune cells. Plasma soluble E-selectin (sE-selectin) levels in *Ldlr⁻/⁻* mice transplanted with either IL-37tg or wild-type (WT) bone marrow were determined just before Western-type diet (WTD)-feeding on regular chow diet, and after eight weeks of WTD-feeding (**a**); The indicated circulating immune cells in whole blood were determined by flow cytometry before (chow) and after three weeks of WTD-feeding (WTD) (**b**–**k**); Moreover, the ratio Ly6C^lo^/Ly6C^hi^ was calculated (**l**); Data are expressed as means ± SEM; *n* = 14–15 per group; In panel 2a the effect of diet was determined using two-way ANOVA; * *p* < 0.05; ** *p* < 0.01; *** *p* < 0.001.

Next, we evaluated the effect of hematopoietic IL-37 expression on inflammatory parameters. WTD-feeding increased plasma sE-selectin as compared to chow-feeding, thereby confirming the induction of low-grade inflammation by the WTD, but hematopoietic IL-37 expression did not reduce plasma sE-selectin (Figure 2a). However, flow cytometry of blood cells showed that hematopoietic IL-37 expression reduced the number of circulating immune cells as evident from reduced circulating granulocytes (Figure 2b), neutrophils (Figure 2c), and eosinophils (Figure 2d) during WTD-feeding. In addition, total T-lymphocytes were reduced during chow- and WTD-feeding (Figure 2e) and cytotoxic T-cells were significantly reduced during chow-feeding (Figure 2f). In contrast, expression of IL-37 did not influence helper T-cells (Figure 2g) and regulatory T-cells (Figure 2h). Although hematopoietic IL-37 expression did not affect the percentage of total circulating monocytes (Figure 2i) or Ly6C^lo^ monocytes (Figure 2j), IL-37 expression reduced the number of newly recruited Ly6C^hi^ monocytes (Figure 2k). The ratio of Ly6C^lo^ versus Ly6C^hi^ monocytes was not significantly increased (Figure 2I). Taken together, these data indicate a reduced inflammatory state by hematopoietic IL-37 expression.

**Figure 3:**
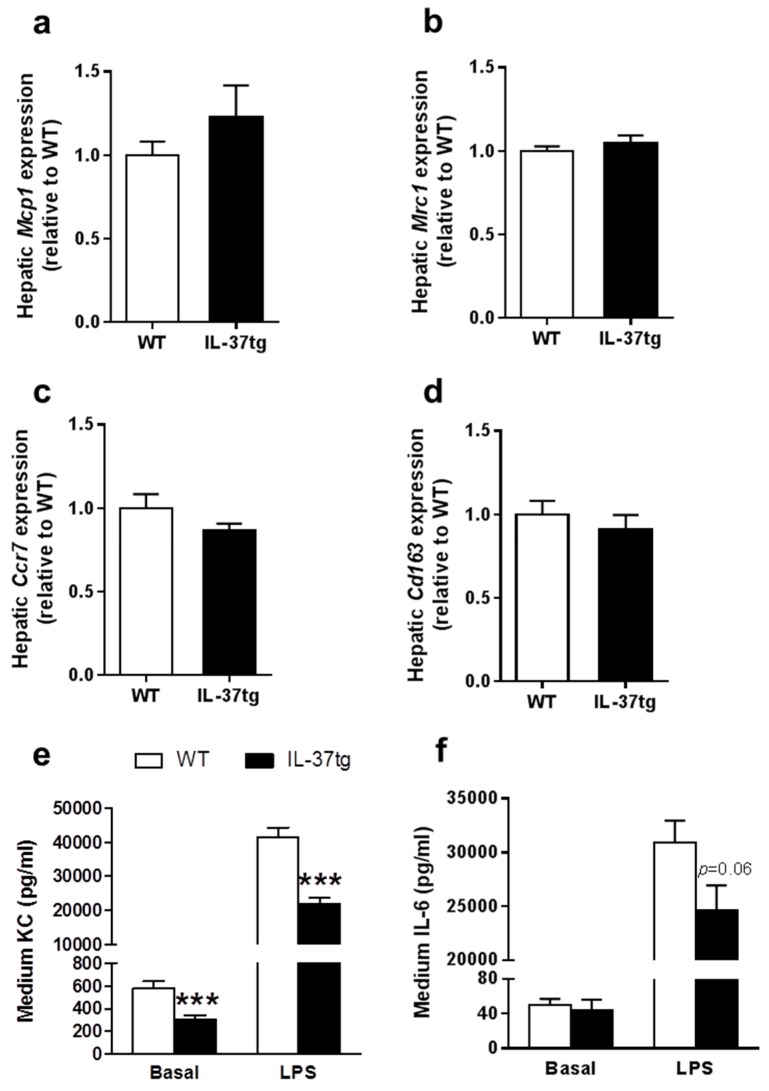
Expression of IL-37 reduces lipopolysaccharide (LPS)-induced cytokine secretion by peritoneal macrophages ex vivo. At the end of the study, after eight weeks of Western-type diet (WTD)-feeding, mice were killed and both liver samples and peritoneal macrophages were isolated. RT-qPCR was used to measure hepatic mRNA expression of the M1 phenotype markers monocyte chemotactic protein 1 (*Mcp1*) (**a**) and mannose receptor C-type 1 (*Mrc1*) (**b**); In addition, the M2 phenotype markers C-C chemokine receptor type 7 (*Ccr7*) (**c**) and cluster of differentiations 163 (*Cd163*) (**d**) were measured (*n* = 15 per group). Peritoneal macrophages were ex vivo stimulated with lipopolysaccharide (LPS; 10 ng·mL⁻^1^) or saline as a control for 24 h; Keratinocyte chemoattractant (KC) (**e**) and IL-6 (**f**) were determined in the medium (*n* = 8 per group). Data are expressed as means ± SEM; *** *p* < 0.001.

Monocytes can differentiate into either classically activated macrophages that secrete pro-inflammatory cytokines (i.e., M1 macrophages), or alternatively activated macrophages that secrete anti-inflammatory cytokines (i.e., M2 macrophages). To evaluate the effects of hematopoietic IL-37 expression on macrophage polarization, we measured mRNA expression of M1 and M2 phenotype markers in the liver, a main organ where macrophages reside [18] and which are replenished by bone marrow transplantation (BMT). Hematopoietic IL-37 expression influenced mRNA levels of neither the M1 phenotype markers monocyte chemotactic protein 1 (*Mcp1*) and mannose receptor C-type 1 (*Mrc1*) nor the M2 phenotype markers C-C chemokine receptor type 7 (*Ccr7*) and cluster of differentiations 163 (*Cd163*) (Figure 3a–d). These data indicate that hematopoietic IL-37 expression did not change macrophage polarization.

We next studied the effect of hematopoietic IL-37 expression on the response of macrophages to inflammatory stimuli. To this end, peritoneal macrophages from IL-37tg bone marrow-transplanted *Ldlr^−/−^* mice were isolated and ex vivo stimulated with LPS. Expression of IL-37 markedly reduced both basal and LPS-stimulated secretion of keratinocyte chemoattractant (KC; i.e., a murine homologue of IL-8) (Figure 3e). Furthermore, IL-37 expression by peritoneal macrophages tended to reduce LPS-induced IL-6 secretion (Figure 3f). These results together indicate that hematopoietic IL-37 expression reduced the inflammatory state and dampened the activation of macrophages.

### 2.3. Hematopoietic IL-37 Expression Does Not Affect Atherosclerosis Development

**Figure 4:**
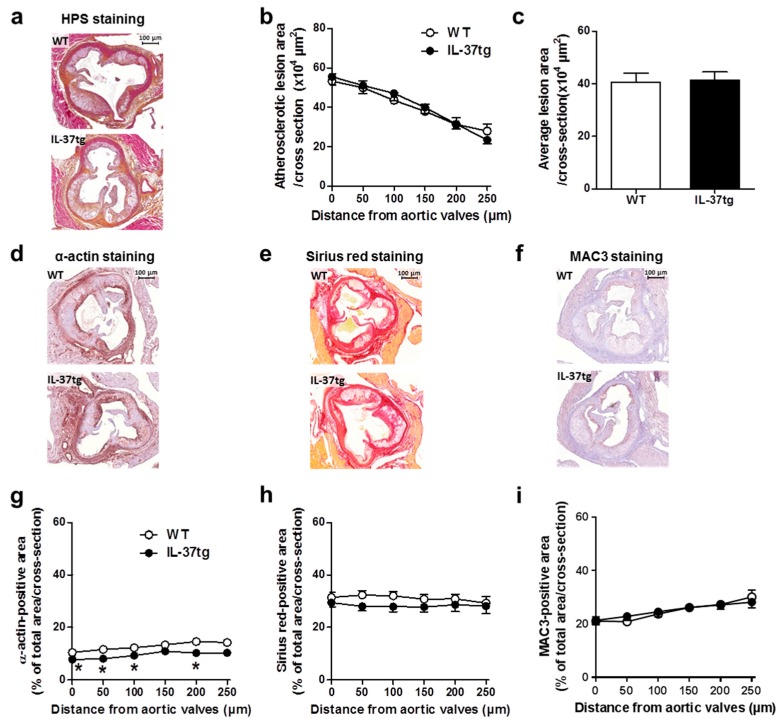
Expression of IL-37 in hematopoietic cells does not affect atherosclerotic lesion development and macrophage content. At the end of the study, after eight weeks of Western-type diet (WTD)-feeding, *Ldlr⁻/⁻* mice transplanted with either IL-37tg or wild-type (WT) bone marrow were killed and slides of the valve area of the aortic root were stained with hematoxylin-phloxine-saffron (HPS) and representative pictures are shown (**a**); (scale bar represents 100 μm.) Lesion area as a function of distance in the aortic root was determined by calculating the lesion area of six consecutive cross-sections starting from the appearance of open aortic valve leaflets (**b**); The average total lesion area of these six cross-sections per mouse was calculated (**c**); Representative pictures of α-actin staining (**d**); Sirius red staining (**e**); and MAC3 (a macrophage-specific antigen) staining (**f**) are presented. The α-actin-positive smooth muscle cell content (**g**); Sirius red-positive collagen content (**h**) and MAC3-positive macrophage content (**i**) were determined. Values are means ± SEM; *n* = 15 per group; * *p* < 0.05.

Finally, we assessed whether the reduced inflammatory state of hematopoietic IL-37 expression would attenuate atherosclerosis development. To this end, we determined the atherosclerotic lesion area in the valve area of the aortic root of the heart as well as atherosclerotic lesion composition after eight weeks of WTD-feeding. Although the atherosclerotic lesion area was unaffected throughout the aortic root of the heart (Figure 4a–c), IL-37 expression reduced the smooth muscle cell content of the atherosclerotic lesions (Figure 4d,g). Both the collagen content (Figure 4e,h) and the macrophage content (Figure 4f,i) remained unchanged between both groups. Taken together, these findings indicate that hematopoietic IL-37 expression marginally affects lesion composition without a strong effect on atherosclerotic lesion size.

## 3. Discussion

IL-37 is an anti-inflammatory component of the immune system. As hematopoietic cell-specific expression of IL-37 is sufficient to protect mice from colitis [16] and recombinant IL-37 treatment reduces atherosclerosis development in mice [17], we investigated in the current study whether transgenic expression of human IL-37 in hematopoietic cells reduces atherosclerosis development characterized by a low-grade inflammatory state. Our data show that hematopoietic expression of IL-37 in atherosclerosis-prone *Ldlr^−/−^* mice reduced the inflammatory state as indicated by lower circulating immune cells in vivo and lower secretion of pro-inflammatory cytokines by peritoneal macrophages ex vivo. However, this reduced inflammatory state did not influence metabolic parameters and was not accompanied by reduced atherosclerosis development or reduced macrophage content of the lesions under these conditions.

Hematopoietic IL-37 expression moderately reduced the inflammatory state of the mice. Although hematopoietic IL-37 expression did not alter the inflammatory marker sE-selectin, it reduced the percentage of circulating immune cells (e.g*.*, neutrophils, eosinophils, Ly6C^hi^ monocytes and total T-cells). These immune cells are attracted to sites of inflammation such as the atherosclerotic lesion and are considered as important contributors to the progression of the atherosclerotic lesion [19,20,21]. Ly6C^hi^ monocytes secrete pro-inflammatory cytokines and reactive oxygen species and, similar to T-cells, promote further lesion progression [19,20]. Neutrophils are the first cells to respond to tissue damage and secrete proteolytic enzymes that will cause the loss of the vascular endothelial structure [21]. Our data corroborate previous findings showing that treatment with recombinant IL-37 also reduced the circulating neutrophil count in disease models of acute lung injury [22] and inflammatory arthritis [23]. Next to these reductions in circulating immune cells, expression of IL-37 also lowered basal and LPS-stimulated cytokine secretion by peritoneal macrophages ex vivo. This is in line with models of colitis where IL-37 expression also reduced the secretion of pro-inflammatory cytokines [16,24]. This, together with the reduction in circulating immune cells, indicates that hematopoietic IL-37 expression reduced the inflammatory state of the mice and dampened the activation of macrophages.

Despite dampening the inflammatory state of the mice, hematopoietic IL-37 expression neither reduced the atherosclerotic lesion size nor the macrophage content of the lesions in the present study. This is in seeming contrast to other recent studies where treatment with recombinant IL-37 reduced atherosclerosis development in streptozotocin-induced diabetic apolipoprotein E-deficient (*Apoe^−/−^*) mice [25], and protected WT mice from endotoxemia-induced cardiac dysfunction [26] and damage-induced myocardial ischaemia/reperfusion injury via a reduction in inflammatory state [27]. Two reasons may account for the lack of effect of hematopoietic IL-37 on atherosclerosis development.

Firstly, it may be possible that hematopoietic IL-37 does not play a role in disease models driven by low-grade systemic inflammation. The hypercholesterolemia that was induced in this study was likely the most important driver of atherosclerosis development and, importantly, was not influenced by IL-37 expression. It is important to realize that IL-37 expression in hematopoietic cells is low under basal conditions and increases during inflammation [28]. In line with this, the protective effect of IL-37 expression was evident in diseases with active chronic or acute inflammation, such as colitis [16], inflammatory arthritis [23] and septic shock [8]. Furthermore, the studies described above studied atherosclerosis and cardiac function in high-inflammatory models, i.e., streptozotocin-induced diabetes [25], endotoxemia [26] and damage-induced ischaemia/reperfusion [27]. In the present study, we show that, under low-grade inflammation, hematopoietic IL-37 expression does not protect from atherosclerosis development. Whether IL-37 protects from other cardiovascular diseases that are characterized by high-grade inflammation (e.g., with myocarditis or endocarditis as the underlying cause) or develop as a comorbidity of high inflammatory diseases (e.g., rheumatoid arthritis) remains a topic for future research.

Alternatively, it may be possible that other IL-37-expressing cells protect from atherosclerosis development, which would imply that systemic IL-37 expression may be required to prevent the development of cardiovascular diseases. In fact, in humans IL-37 is expressed by multiple cell types of e.g., adipose tissue, and IL-37 expression is higher in mature adipocytes as compared to the vascular stromal fraction, i.e., where immune cells are present [11]. Moreover, IL-37 is also expressed by human endothelial cells [10], which play a major role in atherosclerosis progression by functioning as a barrier and mediating the attraction and migration of immune cells into the vessel wall. Also, a mouse study has showed that recombinant IL-37 treatment reduces osteogenic responses to inflammatory stimuli in human aortic valve interstitial cells and reduces the progression of calcific aortic valve disease [29]. The murine studies that used recombinant IL-37 administration had protective effects on atherosclerosis development and cardiac function in highly inflammatory models, indeed suggesting that systemic IL-37 expression is cardioprotective under conditions of high inflammation [25,26,27]. Moreover, a recent study showed that recombinant IL-37 treatment reduced atherosclerosis development and improved plaque composition (i.e., reduced macrophage content and increased collagen and smooth muscle cell content) under low inflammatory conditions [17]. This atheroprotective effect was, at least in part, due to the induction of a regulatory T-cell response. This is in seeming contrast to our study, in which hematopoietic IL-37 expression did not influence the number of regulatory T-cells. It is feasible that the protective effect of IL-37 requires interaction between non-hematopoietic IL-37-expressing cells (e.g., endothelial cells or smooth muscle cells) with immune cells. Alternatively, higher systemic and/or local levels of IL-37 than achieved with hematopoietic IL-37 expression may be required for the atheroprotective effects of IL-37. Likely, recombinant IL-37 treatment sufficiently increases IL-37 levels. Collectively, these studies indicate that IL-37 production by cell types other than hematopoietic cells, the interaction between various IL-37-expressing cells and/or higher local levels are required for the atheroprotective effect of IL-37.

## 4. Materials and Methods

### 4.1. Animals

Homozygous *Ldlr^−/−^* mice (C57Bl/6J background) were obtained from the Jackson Laboratory (Bar Harbor, ME, USA). Mice were housed under standard conditions in conventional cages in a temperature-controlled room with a 12-h light/dark cycle and ad libitum access to food and water. To induce bone marrow aplasia, female *Ldlr^−/−^* recipient mice (eight weeks of age) were exposed to a single dose of 8 Gy using an Orthovolt X-ray machine (CAM/P10008, 081806, Varian Medical Systems, Houten, The Netherlands). The day thereafter, the irradiated recipient *Ldlr^−/−^* mice received an intravenous injection via the tail vein with 1.2 × 10^6^ bone marrow cells isolated from donor IL-37tg [8] or control C57BL/6 WT female mice. In addition, 0.3 × 10^6^ freshly isolated splenic cells from *Rag1^−/−^* female mice, which do not have T- and B-cells, were also added to the injection mixture to ensure the presence of circulating myeloid cells during the first weeks after BMT. All mice received antibiotics-water (0.13 mg·kg^−1^·day^−1^ Ciprofloxacin, 0.105 mg·kg^−1^·day^−1^ Polymyxin B, 0.15 mg·kg^−1^·day^−1^ Amfotericine B) from one day before until four weeks after the bone marrow transplantation (BMT). After nine weeks of recovery on a chow diet, mice were fed a WTD, containing 15% (*w*/*w*) cocoa butter, 1% (*w*/*w*) corn oil and 1% (*w*/*w*) cholesterol (AB diets, Woerden, The Netherlands) for eight weeks. Body weight was monitored weekly, and food intake per cage (4–5 mice per cage) twice a week. All animal experiments were performed in accordance with the Institute for Laboratory Animal Research Guide for the Care and Use of Laboratory Animals and received approval from the local animal ethical committee (DEC 12157, approved on 15 August 2012, Leiden University Medical Center, Leiden, The Netherlands).

### 4.2. Assessment of Successful Bone Marrow Reconstitution

At the end of the study, bone marrow was isolated from the tibia and hematologic chimerism was determined using genomic DNA by polymerase chain reaction (PCR) at 17 weeks after BMT, which was isolated using the Gentra Pure Blood Kit (Qiagen, Venlo, The Netherlands). The relative presence of IL-37 in bone marrow was assessed using primers for human IL-37 (forward: 5′-CGATTCTCCTGGGGGTCTCTA-3′; reverse: 5′-CGGCGTGCTGATTCCTTTTG-3′).

### 4.3. Plasma Lipid and Systemic Inflammation Analysis

Blood was drawn from the tail vein of 4 h fasted mice at the indicated time points. After eight weeks of WTD, unfasted blood samples were collected via orbital exsanguination in ethylenediaminetetraacetic acid (EDTA)-coated tubes. Plasma from all samples was isolated by centrifugation and assayed for total cholesterol, triglycerides (Roche Diagnostics, Mannheim, Germany) and phospholipids (Instruchemie, Delfzijl, The Netherlands) using commercially available enzymatic colorimetric kits. Plasma levels of soluble E-selectin (sE-selectin) were determined using murine E-selectin ELISA kit (R&D, Minneapolis, MN, USA). All assays were preformed according to the manufacturers’ protocols.

### 4.4. Flow Cytometry

Blood was collected before the start (“chow”) and during the WTD-feeding (“WTD”) in EDTA-coated tubes. After lysing the total erythrocytes, subsets of T-lymphocytes, monocytes and granulocytes were assessed by standard fluorescence-activated cell sorting (FACS) analysis. Cells were stained using fluorochrome-conjugated monoclonal antibodies for CD4 (eBioscience, San Diego, CA, USA), CD8 (Biolegend, San Diego, CA, USA), CD11b, CD25, Ly6G clone 1A8 and Ly6C (all BD Pharmingen, San Diego, CA, USA). Intracellular FoxP3 staining was performed using a FoxP3 staining set (eBioscience). Data were acquired on a FACSAria or a FACSCanto II (BD Biosciences, San Diego, CA, USA) and analyzed using FlowJo software (Treestar, Ashland, OR, USA). Data are presented as percentage of live cells.

### 4.5. *Ex Vivo* Stimulation of Peritoneal Macrophages

At the end of the study peritoneal macrophages were isolated and stimulated ex vivo with 10 ng·mL^−1^ *Escherichia coli* lipopolysaccharide (*E. coli* LPS; serotype O55:B5, Sigma Aldrich, St. Louis, MO, USA), which was further purified as described in [30] or kept in saline as a control for 24 h. Keratinocyte chemoattractant (KC) and IL-6 concentrations were determined in the medium by commercial ELISA kits (Biosource, Camarillo, CA, USA) according to the instructions of the manufacturer.

### 4.6. Gene Expression Analysis

RNA was extracted from snap-frozen mouse liver samples (approx. 30 mg) using a tripure RNA isolation reagent (Roche) according to the manufacturer’s protocol. Total RNA (1 μg) was reverse transcribed using Moloney murine leukemia virus (M-MLV) reverse transcriptase (Promega) for qRT-PCR according to the manufacturer’s instructions to produce cDNA. mRNA expression was normalized to β2-microglobulin mRNA expression and expressed relative to WT mice using the ΔΔ*C*_t_ method. The primer sequences used are listed in Table 1 below.

### 4.7. Atherosclerosis Development

Hearts were collected and fixed in phosphate-buffered 4% formaldehyde, embedded in paraffin and cross-sectioned (5 μm) throughout the aortic root area, starting from the appearance of open aortic valve leaflets. Per mouse, six sections with 50 μm intervals were used for atherosclerosis quantification. Sections were stained with hematoxylin–phloxine–saffron for histological analysis. The macrophage area was determined using rat anti-mouse antibody MAC3 (1:1000; BD Pharmingen, San Diego, CA, USA). The smooth muscle cell area was quantified using monoclonal mouse antibody M0851 (1:800; Dako, Heverlee, The Netherlands) against smooth muscle cell actin. The collagen area was determined using Sirius Red staining. The lesion area and composition were quantified using ImageJ Software.

### 4.8. Statistical Analysis

The differences between genotypes were determined using a two-tailed Student T-Test. Specifically for Figure 2a, differences between genotype and type of diet were determined using two-way ANOVA. Differences at probability values less than 0.05 were considered statistically significant. Data are presented as means ± standard error of the mean (SEM). All statistical analyses were performed with the SPSS 20.0 software package for Windows (SPSS, Chicago, IL, USA).

## 5. Conclusions

In conclusion, hematopoietic IL-37 expression in hyperlipidemic *Ldlr^−/−^* mice with low-grade systemic inflammation reduced the number of circulating immune cells. In addition, the secretion of LPS-stimulated pro-inflammatory cytokines by peritoneal macrophages ex vivo was decreased. However, hematopoietic IL-37 did not influence hypercholesterolemia and atherosclerosis development.

## Figures and Tables

**Table 1:** Primer sequences.

Gene	Forward Primer	Reverse Primer
*Ccr7*	ATGGACCCAGGGAAACCCAGGAA	CAGTATCACCAGCCCGTTGCCG
*Cd163*	CTCAGGAAACCAATCCCAGA	CAAGAGCCCTCGTGGTAGAC
*β2-microglobulin*	TGACCGGCTTGTATGCTATC	CAGTGTGAGCCAGGATATAG
*Mcp1*	GCATCTGCCCTAAGGTCTTCA	TTCACTGTCACACTGGTCACTCCTA
*Mrc1*	GAGAGCCAAGCCATGAGA	GTCTGCACCCTCCGGTAC

*Ccr7*, C-C chemokine receptor type 7; *Cd163*, cluster of differentiation 163; *Mcp1*, monocyte chemotactic protein 1; *Mrc1*, mannose receptor C-type 1.

## Abbreviations

BMT	Bone marrow transplantation
IL	Interleukin
KC	Keratinocyte chemoattractant
LDL(r)	Low-density lipoprotein (receptor)
LPS	Lipopolysaccharide
sE-selectin	Soluble E-selectin
Tg	Transgenic
WT	Wild-type
WTD	Western-type diet
